# Robust hand tracking for surgical telestration

**DOI:** 10.1007/s11548-022-02637-9

**Published:** 2022-05-27

**Authors:** Lucas-Raphael Müller, Jens Petersen, Amine Yamlahi, Philipp Wise, Tim J. Adler, Alexander Seitel, Karl-Friedrich Kowalewski, Beat Müller, Hannes Kenngott, Felix Nickel, Lena Maier-Hein

**Affiliations:** 1 Intelligent Medical Systems (IMSY), German Cancer Research Center (DKFZ), Heidelberg, Germany; 2 Division of Medical Image Computing (MIC), German Cancer Research Center (DKFZ), Heidelberg, Germany; 3 Faculty of Mathematics and Computer Science, Heidelberg University, Heidelberg, Germany; 4 Department for General, Visceral and Transplantation Surgery, Mannheim University Hospital, Heidelberg, Germany; 5 Department of Urology and Urosurgery, Medical Faculty Mannheim, Heidelberg University Hospital, Heidelberg, Germany; 6 Medical Faculty, Heidelberg University, Heidelberg, Germany

**Keywords:** Hand tracking, Telestration, Surgical data science, Computer vision, Deep learning

## Abstract

****Purpose**:**

As human failure has been shown to be one primary cause for post-operative death, surgical training is of the utmost socioeconomic importance. In this context, the concept of surgical telestration has been introduced to enable experienced surgeons to efficiently and effectively mentor trainees in an intuitive way. While previous approaches to telestration have concentrated on overlaying drawings on surgical videos, we explore the augmented reality (AR) visualization of surgical hands to imitate the direct interaction with the situs.

****Methods**:**

We present a real-time hand tracking pipeline specifically designed for the application of surgical telestration. It comprises three modules, dedicated to (1) the coarse localization of the expert’s hand and the subsequent (2) segmentation of the hand for AR visualization in the field of view of the trainee and (3) regression of keypoints making up the hand’s skeleton. The semantic representation is obtained to offer the ability for structured reporting of the motions performed as part of the teaching.

****Results**:**

According to a comprehensive validation based on a large data set comprising more than 14,000 annotated images with varying application-relevant conditions, our algorithm enables real-time hand tracking and is sufficiently accurate for the task of surgical telestration. In a retrospective validation study, a mean detection accuracy of 98%, a mean keypoint regression accuracy of 10.0 px and a mean Dice Similarity Coefficient of 0.95 were achieved. In a prospective validation study, it showed uncompromised performance when the sensor, operator or gesture varied.

****Conclusion**:**

Due to its high accuracy and fast inference time, our neural network-based approach to hand tracking is well suited for an AR approach to surgical telestration. Future work should be directed to evaluating the clinical value of the approach.

**Supplementary Information:**

The online version contains supplementary material available at 10.1007/s11548-022-02637-9.

## Introduction

**Fig. 1:**
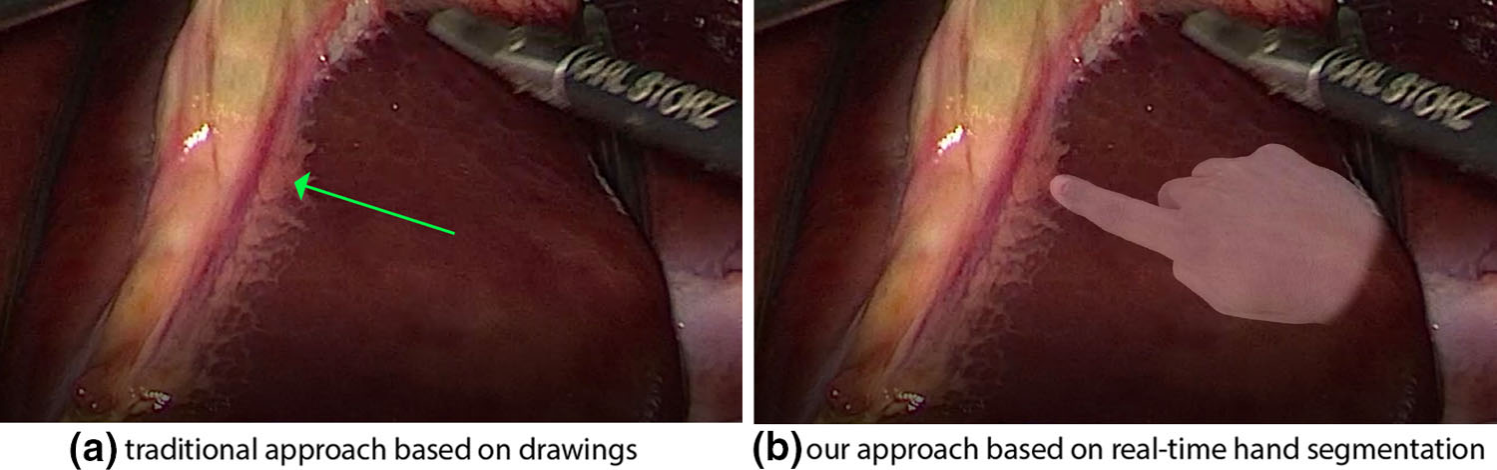
Our telestration approach compared to the state of the art. **a** Previous approaches to surgical telestration rely on overlaying drawings on laparoscopic videos, while our concept is based on **b** the augmented reality (AR) visualization of the expert surgeon’s hand

Death within 30 days after surgery has recently been found to be the third-leading contributor to death worldwide [1]. As a large portion of deaths can be attributed to human failure, surgical training is of the utmost socioeconomic importance. In this context, the concept of surgical telestration has been introduced to enable experienced surgeons to efficiently and intuitively mentor trainees during surgical training [2]. In the context of laparoscopic surgery, the key idea is to offer the mentor the ability to highlight/point at anatomical structures within the surgical video to guide the trainee during the surgery. Intuitively one would expect senior surgeons to physically point at anatomy directly on the display, but this comes with several restrictions regarding hardware setup, surgical workflow as well as sterilization requirements, because the mentor would need to be able to touch the monitor that the trainee is looking at. As a result, this approach is almost never used in practice. More recent computer-assisted alternatives for surgical telestration [3–5] typically require the mentor to operate on a separate computer system to draw simple overlays (e.g., lines, circles) onto the video seen by the trainee (Fig. 1). While this approach can even be used for remote training, it is less intuitive, slower and challenging to implement in the presence of high organ deformation.

**Fig. 2:**
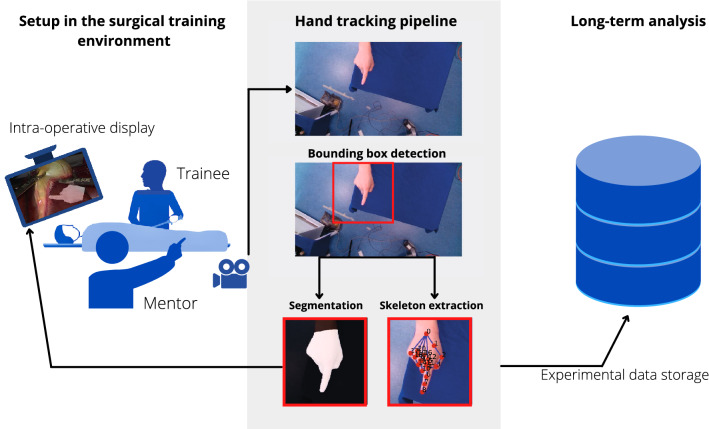
Concept overview. Our approach to surgical telestration relies on a camera that continuously captures a hand of the mentor who observes the operation either on-site or remotely. The camera data are processed by a two-stage neural network, which outputs both the skeleton (represented by 21 keypoints) and the segmented hand. The hand segmentation is overlaid on the surgical screen for intuitive coaching, while the skeleton representation is stored for long-term analysis

To exploit the benefits of both approaches, we investigate a concept in which the hands of the experienced surgeon are continuously monitored and transferred as an augmented reality (AR) overlay onto the laparoscopic video (patent pending [6]) (Figs. 1 and 2). The mentor is then able to be in direct interaction with the surgical trainee and provide intuitive guidance via hand gestures seen by the trainee on the surgical video. The concept is also applicable in remote settings because the mentor does not need to be present in the OR.

Key to the performance of this new concept of surgical telestration is an automatic accurate, robust and fast hand tracking. In this paper, we present the first hand tracking pipeline specifically designed for the application of surgical telestration (Fig. 2). In contrast to related approaches in the surgical domain, that focus on coarse hand and object tracking, localization and pose estimation [7–9], our method simultaneously outputs a fine-grained hand segmentation as an important prerequisite for the AR overlay. We further perform a comprehensive validation of the method based on a surgical data set of unprecedented size, comprising more than 14,000 images and reflecting application-specific challenges, such as changes in skin and surgical glove color.

## Material and methods

Our approach to surgical telestration is depicted in Fig. 2. A camera captures the hands of the mentor who observes the (training) operation. The camera data are processed by a two-stage neural network, which outputs (1) a skeleton representation comprising positions of 21 keypoints as a semantic representation of the hand and (2) a segmentation of the hand for AR visualization in the field of view of the trainee. The skeleton representation is used as an auxiliary task for the hand segmentation as well as for long-term analysis of the application. In this paper, we focus on the hand tracking module, which is key to the performance of our approach. The underlying data set is described in “Data set” section. Our approach is based on the coarse localization of the hand via a bounding box (“Real-time hand localization” section) and the subsequent extraction of the skeleton (“Real-time skeleton tracking” section) and the hand segmentation (“Real-time hand segmentation” section).

### Data set

#### Data acquisition

The data for development and initial validation of the hand tracking methodology were acquired at the Heidelberg University Hospital using a Real Sense D435i camera (Intel; Santa Clara, USA) in a surgical training setting. We acquired a total of 14,102 images on 66 days between March 2020 and April 2021, featuring a variety of different hand poses, lighting conditions as well as hand and glove colors. Our data set comprises approx. 46% light skin, 22% blue glove, 11% white glove, 11% green glove, 8% brown glove and 2% dark skin. As the telestration concept should also be applicable in settings with unpredictable background, we varied the latter, specifically with respect to the objects present. While we allowed for multiple hands to be present in the field of view of the camera, our concept assumes one *primary hand* used for telestration by the mentor.

#### Data annotation

**Fig. 3:**
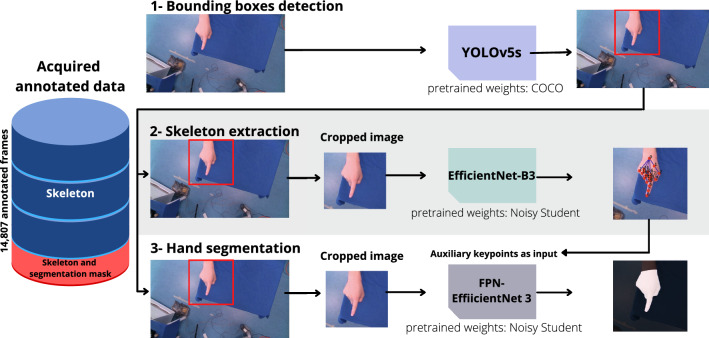
Overview of the models used for real-time hand tracking. Our approach comprises three core components. (1) a bounding box module using the YOLOv5s architecture, (2) a skeleton tracking module using an EfficientNet B3 and (3) a segmentation module using a FPN-EfficientNet B1. (2) and (3) operate on images cropped to the respective bounding boxes (see “Real-time hand localization”, “Real-time skeleton tracking”, “Real-time hand segmentation” section)

In the acquired images, a medical expert annotated the primary hand of the expert by setting 21 keypoints representing the skeleton of the hand, as suggested in [10] and shown in Figs. 2 and 3. Additional metadata annotations include handedness (left, right), the skin (light, dark) or glove color (brown, blue, green, white) in the presence of the latter. A subset of 499 images^1^ was then extracted, and a medical expert complemented the skeleton annotations with segmentations of the entire hand, as illustrated in Figs. 2 and 3.

#### Data set split

We split our data into a proportion of 80:20 for training (including hyperparameter tuning on a validation set) and assessment of performance. We note that videos taken on the same day are not necessarily independent due to comparable hardware setups. Therefore, we ensured that no data from the same day are present in both training and test set. To prevent data leakage, the segmentation train/test set is a subset of the corresponding keypoint train/test set. To achieve an adequate train/test split, we sampled days for the test set randomly, until a proportion of 20% was reached. We repeated this procedure once for the part of the data set with segmentation annotation and once for the part without. As we expect the measurement days to be a major contributor to the variance in the data set, we want to make sure that a sufficient number of measurement days in the test set. To guarantee this, we excluded the three measurement days with the most measurements from the test set. To split the training data into a training and validation set, a similar procedure was followed, but with only 10% of training data used for the validation set. For skeleton tracking, this resulted in a data set size of 11,541 as training set (including validation) and 2561 as the test set. For the segmentation task, a total of 395 images served as training set (including validation), the remaining 104 images served as test set. The validation data set was used to optimize the processing pipeline (see A.1); the test data set was used for performance assessment.

### Real-time hand localization

Inspired by the MediaPipe model [10], we use a bounding box detection step prior to both skeleton tracking as well as segmentation. Our reference bounding boxes were derived from the skeleton points by first constructing a tight bounding box that encloses all skeleton points and then enlarging this box by a factor of two and squaring it. Using the cropped image as input for the skeleton extraction and segmentation ensures a relatively constant size of the hand and enables us to phrase the skeleton extraction task as a regression task based on the 21 keypoints. While MediaPipe uses a single shot palm detector [10], we apply YOLOv5s [11] as our detection model to predict the bounding boxes as we identified YOLOv5s being a good compromise between accuracy and speed [12]. The predicted boxes are post-processed using the Non-Maximum-Suppression (NMS) algorithm with an Intersection over Union (IoU) threshold of 0.5. Unlike Mediapipe, where bounding boxes are only inferred if a detection is considered lost, we employ the bounding box model continuously. This is possible due to the short inference times of the YOLOv5s model in conjunction with our hardware setup. The box with the highest confidence score is used in the downstream tasks.

We use the training procedure as presented in the official implementation [11] with a stochastic gradient descent (SGD) optimizer, learning rate of 0.01 and binary cross-entropy as the loss function. The augmentations used for training stem from the official implementation, namely: hue, saturation, value, and mosaic augmentations, and horizontal flips. We save the weights based on the epoch with the best mean average precision (mAP).

### Real-time skeleton tracking

Our skeleton tracking architecture operates on the cropped images generated by the bounding box model. We use an EfficientNet B3 [13, 14] loaded with Noisy Student pretrained weights [15] as the backbone for our regression model with an L1 loss for optimization. The model is trained using the Adam optimizer with an automatically set learning rate [16]. We save the model weights based on the mean regression accuracy on the validation set, which is the mean Euclidean distance between annotated reference and model prediction of the skeleton joints. During training, we use random offsets and alter the size of the reference bounding box to account for the fact that the regression and segmentation models will not be provided with perfect bounding boxes at inference time. In addition, we use the following augmentations implemented in the Albumentations library [17] namely: brightness and contrast, RGB channel shuffle, RGB shift, hue saturation, Gaussian noise, blur, rotation. The aforementioned augmentations are activated with per sample probability $p=0.15$.

### Real-time hand segmentation

#### Baseline model

As for the hand skeleton, we utilize cropped images based on bounding boxes for the segmentation. Our segmentation model uses a Feature Pyramid Networks FPN [18] with an EfficientNet B3 encoder loaded with Noisy Student pretrained weights [15] as a backbone and is trained by optimizing the binary cross-entropy loss using an adam optimizer with a learning rate of $10^{-4}$. We utilize the same augmentations in the Albumentations [17] as for the skeleton training.

#### Model with auxiliary task

In a variant of this approach, we use the skeleton tracking task as an auxiliary task for our model. To this end, the 21 keypoints regressed by our skeleton tracking model are used as additional input (one channel per keypoint) of the hand tracking module. Each channel contains a two-dimensional isotropic Gaussian centered at the keypoint location and a standard deviation of 5 px. To account for the different input channels, while being able to utilize pretraining, we add three CNN layers that merge the RGB with the Gaussian input prior to the actual backbone.

**Fig. 4:**
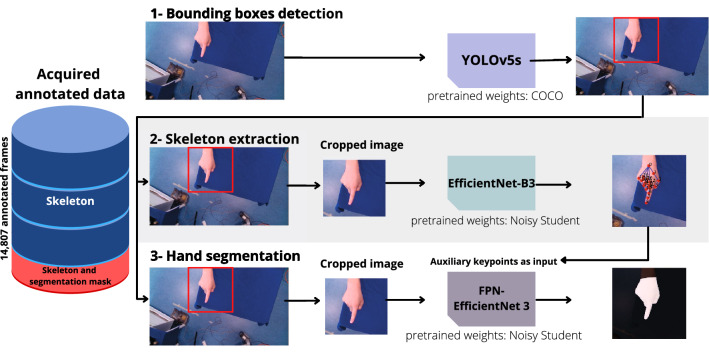
Representative results for a diverse set of gestures. The outputs of the three models for bounding box prediction (top) as well as skeleton tracking and segmentation (bottom) are shown

The primary purpose of our study was to assess the accuracy, robustness and speed of our hand tracking pipeline in the surgical training setting. Specifically, we investigated the regression accuracy for the keypoints making up the skeleton of the hand (“Real-time skeleton tracking” section), as well as the segmentation accuracy (“Real-time hand segmentation” section) set whose distribution was similar to that of the training set. In a second, prospective study, we assessed the generalization capabilities of our method, by including mentors, gestures and cameras that were not part of the training data (“Assessment of generalization capabilities” section).

#### Assessment of speed and accuracy

As an initial retrospective validation of our method, we determined the speed and accuracy for the skeleton tracking and hand segmentation using the test set of the data set described in “Data set” section. The workstation for the assessment of the inference time was equipped with a Nvidia Geforce RTX 3090 and an AMD Ryzen 9 3900X 12-Core Processor.

### Real-time skeleton tracking

**Fig. 5:**
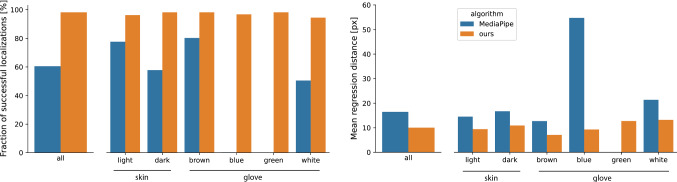
Skeleton tracking performance for our method (orange) vs. MediaPipe (blue) as the baseline. Fraction of successful localizations (left) and mean regression distance (right) for successful localizations and validated with respect to the different hand properties. Note that for MediaPipe there are only very few successful localizations for blue gloves and none for green ones

**Fig. 6:**
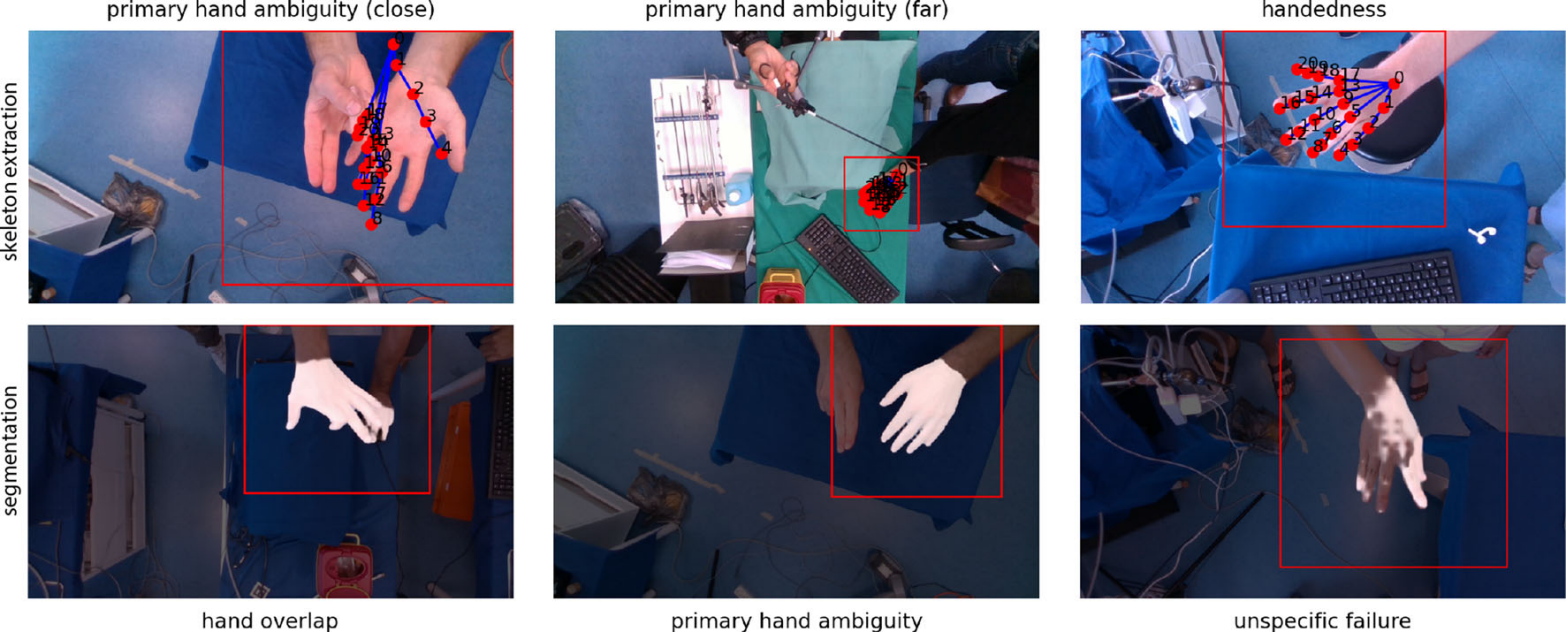
Representative failure cases of the skeleton extraction model (top row) and the segmentation model (bottom row)

#### Experiments

To assess the skeleton tracking accuracy, we determined descriptive statistics over the mean keypoint distance on the test set, using MediaPipe [10] as our baseline, because it is widely used and was specifically developed for integration of hand tracking in third-party applications. The hierarchical structure of the data was respected by aggregating over individual frames of one acquisition day before performing the aggregation over all acquisition days. As the MediaPipe method struggled in the presence of dark skin and gloves, we (1) additionally assessed the performance grouped by skin/glove color and (2) divided the performance assessment into the steps of successful hand detection and the keypoint regression performance. We approximated a successful hand detection by comparing the center of gravity of the regressed skeleton joints with the corresponding center of gravity of the reference skeleton joints. A distance below 100 px was regarded as a match. To enable a fair comparison, we compensated for the fact that MediaPipe has no notion of a *primary hand*. To this end, we chose the bounding box out of the four bounding boxes with the highest confidence, that was closest to the reference bounding box. The analysis of the regression performance was then only computed for successful localizations. Note that there is a tradeoff between regression accuracy and detection accuracy depending on the threshold.

#### Results

The results of our method compared to the MediaPipe method are shown in Fig. 5. We achieved a successful localization rate of 98% and a corresponding mean keypoint regression accuracy of 10.0 px, averaged over all samples (after hierarchical aggregation). Compared to the baseline method with a successful localization rate of 60% and keypoint regression accuracy of 16.5 px, this corresponds to a relative improvement of 63%(detection) and 39%(regression). The mean interquartile range (IQR) per video was 4.5 px (light skin), 3.6 px (dark skin), 3.8 px (brown glove), 4.2 px (blue glove), 2.7 px (green glove) and 6.5 px (white glove) for our method (averaged over all videos). On average, the IQR was three times higher for MediaPipe. Notably, the baseline method failed almost completely in the presence of colored (blue, green) gloves, while our method achieved relatively high detection performance despite the low number of training samples for these classes. Representative examples for our method are shown in Fig. 4. Failure cases of our method occurred primarily due to ambiguity in the identification of the primary hand and handedness as illustrated in Fig. 6. The inference time was approximately 16 ms/frame for bounding box detection and 12 ms/frame for skeleton tracking.

### Real-time hand segmentation

#### Experiments

To assess the hand segmentation performance, we determined the Dice Similarity Coefficient (DSC) between the algorithm output and the corresponding reference annotation. The hierarchical structure of the data was respected as described in the previous paragraph. We investigated our method as described above using the sole RGB images as input (1) and (2) using the hand skeleton as additional input (“Real-time skeleton tracking”).

#### Results

The DSC averaged over all samples (after hierarchical aggregation) was 0.95 (interquartile range (IQR): 0.02) both for our method with and without leveraging keypoints as auxiliary input. Representative examples are shown in Fig. 4. Failure cases of our method occurred primarily due to primary hand ambiguity (as for skeleton tracking) and multiple hand overlap, as illustrated in Fig. 6. The inference time was approximately 65 ms and 11 ms per frame for segmentation with and without auxiliary skeleton input, respectively.

### Assessment of generalization capabilities

#### Experiments

To assess the generalization capabilities of our method with respect to new mentors, gestures and cameras, we performed a prospective quantitative validation, for which a total of 705 video frames from three different mentors, two different cameras and two different gestures were acquired and annotated.^2^ None of the subjects and only one of the cameras had been part of the data set used for method development. For each subject and camera, we recorded two surgical gestures in a surgical training setting, namely *pointing along a circular shape* and *pinching*. This resulted in 12 video clips comprising a total of 705 frames. In all frames, the skeleton and outline of the primary hand were annotated, resulting in a test set both for the skeleton tracking as well as the hand segmentation.

#### Results

**Fig. 7:**
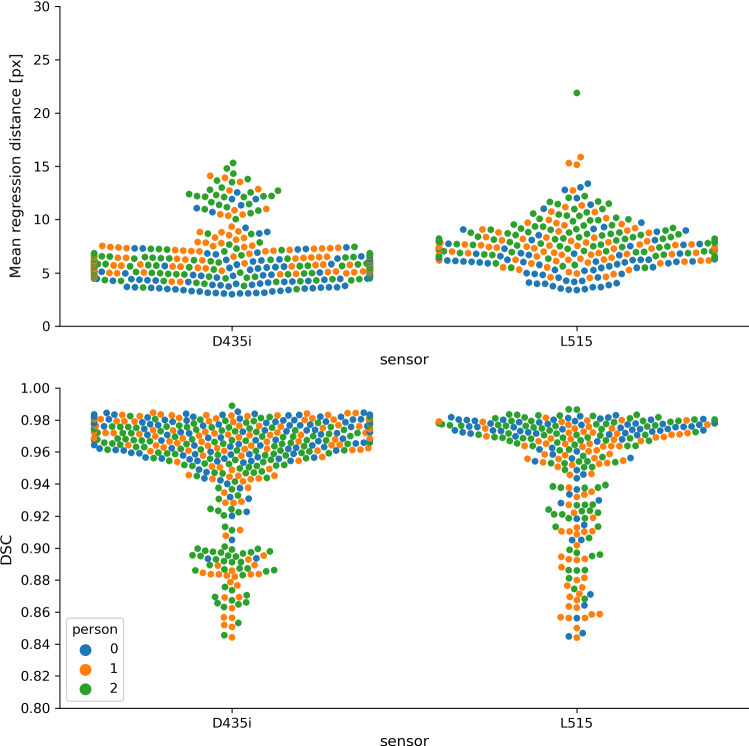
Results of the prospective validation study. Skeleton tracking performance (upper row), quantified by mean regression distance and hand tracking performance (lower row), quantified by the dice similarity coefficient (DSC) are shown for the camera used in the training data set (D435i) as well as a previously unseen camera (L515). Each color corresponds to a different mentor. No notable differences were obtained for the different gestures

The results for skeleton tracking and segmentation are consistent with those obtained in the retrospective study, as shown in Fig. 7, where we used the segmentation model with aux. keypoints. Only minor differences in performance for different sensor, mentors or gestures could be observed, namely 6.2 px vs. 7.6 px mean regression distance and 0.95 vs. 0.95 DSC for the previous vs. unseen sensor.

## Discussion

Surgical telestration is evolving as a promising tool in surgical education and telesurgery. To date, however, the means for communicating instructions to the trainee have been rather limited and focused on manual drawing, e.g., as illustrated in Fig. 1a. To address this bottleneck, this work is the first to explore the automatic tracking of the mentor’s hand with the goal of rendering it directly in the field of view of the trainee.

The potential advantages of this approach are manifold.

- *Real-time feedback:* The mentor does not lose time generating the drawings.
- *Complexity of instructions:* As the mentor can use their own hands, arbitrarily complex gestures can be transmitted to the trainee.
- *High robustness:* There is no need for the error-prone task of dynamically updating the drawings within the field of view of the trainee in the presence of organ deformation.

According to a comprehensive validation study involving 14,102 annotated images acquired in a surgical training setting, our method is highly accurate, yielding a hand detection accuracy of over 98% and a keypoint regression accuracy of 10.0 px. Our approach to skeleton extraction also outperformed the widely used MediaPipe [10] by a large margin, which can mainly be attributed to the presence of colored gloves in our application setting. We speculate that our performance advantage stems from the combination of the large dataset and the introduction of recent state-of-the-art models in our pipeline. To which extent the individual parts contribute to the overall performance remains to be determined once a direct comparison with state-of-the-art approaches is feasible. It should be noted in this context, that it is not straightforward to reproduce a training of MediaPipe on our own data because (1) the official repository is intended for inference and (2) the authors specifically attribute part of their performance to the presence of simulated data stemming from a commercial hand model. To the best of our knowledge, open-source work that is related to MediaPipe only utilizes the MediaPipe pipeline for downstream tasks, i.e., without retraining; we are not aware of open-source code that implements the full pipeline which would allow to compare the performance of this algorithm on our data. We did compensate for the effect that MediaPipe was not trained to detect only a primary hand as we chose the hand that was closest to the reference hand. While the detection threshold of 100 px could be considered arbitrary, we observed no major effect when changing it reasonably.^3^

We obtained a high segmentation accuracy (DSC 0.95) despite the availability of only 395 training images. Using auxiliary keypoints did improve performance in some challenging cases but did not yield to a substantial boost in mean accuracy. Our prospective quantitative robustness validation did not show major degradation of performance when (1) an unseen sensor recorded the frames or (2) unseen mentors were recorded.

A limitation of our work could be seen in the fact that we did not leverage temporal data for our hand tracking task. This is in line with previous work in the field of surgical video analysis [19, 20], but should be investigated in future work. In particular, this holds true for explicit tracking in long video sequences in which the identities should be preserved (e.g., Zhang et al. output processed bounding box tracking [7], Louis et al. incorporate temporal information in the model input [9]). Similarly, inclusion of depth information is a promising next step that would, however, put restrictions on the camera used. Furthermore, incorporating synthetic data is an interesting approach to increase the data set diversity [8].

It should be noted that the DSC, while being commonly reported in the field of medical imaging [21], might not be the optimal choice to assess the performance of our segmentation model in the present environment. We found that the part of the segmentation at the position of the wrist is sometimes ambiguous and was not always consistently labelled by our annotators. In our application of surgical telestration, the end of the hand towards the wrist is not as important as the accurate prediction at the edges of the fingers of the mentor. Therefore, the DSC could be considered a lower bound to our performance, but a study on inter-rated variability needs to confirm this hypothesis.

Hand tracking is a highly relevant task that has been tackled by many authors from various fields [22–25]. However, our validation results on the popular Google MediaPipe [10] demonstrate that the surgical domain comes with unique challenges (e.g., surgical gloves) that have not been addressed by current work. Prior work in the specific field of surgical data science is only recently upcoming. It has focused on bounding box tracking and hand pose estimation based on video data [7–9], alternative approaches utilize external (wearable) sensors [26]. In this paper, we went beyond the state of the art by (1) providing a complete pipeline for surgical hand localization, skeleton extraction and segmentation and (2) validating the benefit of our approach in the specific application context of surgical telestration.

With a frame rate of 20 fps and 11 fps for our entire pipeline with and without using the skeleton extraction as auxiliary task for the segmentation, our pipeline allows for fast inference. As a next step, we will not only optimize the segmentation processing with auxiliary task to achieve real-time performance but also determine the latency of our approach in a realistic application scenario including the actual rendering of the hand. Note in this context that we have decided to first apply the proposed concept in a surgical training environment. Once the approach has been optimized in this setting, we can move on to the more complex operating room.

In conclusion, we (1) presented a novel concept to surgical telestration that tracks the mentor’s hand to overcome major challenges associated with state-of-the-art telestration concepts and (2) validated the performance of its core component based on a very large application-specific data set. Owing to the near-optimal performance and fast inference times obtained for the hand tracking pipeline in our validation study, we are now planning to evaluate the clinical benefit of the hand tracking in a real surgical telestration setting.

## Supplementary Information

Below is the link to the electronic supplementary material.

Supplementary file 1 (mp4 2767 KB)

Supplementary file 2 (mp4 2012 KB)

Supplementary file 3 (pdf 1842 KB)

## Footnotes

1 We aimed for 500 images, but obtained one corrupt segmentation

2 Due to the mounting system of the camera the videos were recorded sequentially.

3 We note that during the review process a new version of MediaPipe was released. The results presented in this work are based on MediaPipe version prior to v0.8.9. Preliminary results on version v0.8.9 show improved although not comparable performance, the drop in performance for blue and green colored gloves persisted.
