# Correction to: Robust hand tracking for surgical telestration

**DOI:** 10.1007/s11548-022-02702-3

**Published:** 2022-07-08

**Authors:** Lucas-Raphael Müller, Jens Petersen, Amine Yamlahi, Philipp Wise, Tim J. Adler, Alexander Seitel, Karl-Friedrich Kowalewski, Beat Müller, Hannes Kenngott, Felix Nickel, Lena Maier-Hein

**Affiliations:** 1 Intelligent Medical Systems (IMSY), German Cancer Research Center (DKFZ), Heidelberg, Germany; 2 Division of Medical Image Computing (MIC), German Cancer Research Center (DKFZ), Heidelberg, Germany; 3 Faculty of Mathematics and Computer Science, Heidelberg University, Heidelberg, Germany; 4 Department for General, Visceral and Transplantation Surgery, Mannheim University Hospital, Heidelberg, Germany; 5 Department of Urology and Urosurgery, Medical Faculty Mannheim, Heidelberg University Hospital, Heidelberg, Germany; 6 Medical Faculty, Heidelberg University, Heidelberg, Germany

## Correction to: International Journal of Computer Assisted Radiology and Surgery 10.1007/s11548-022-02637-9

The original version of this article unfortunately contained a mistake. The affiliation details for Author Lena Maier-Hein were incorrectly given as:

Department of Urology and Urosurgery, Medical Faculty Mannheim, Heidelberg University Hospital, Heidelberg, Germany

but should have been:

Medical Faculty, Heidelberg University, Heidelberg, Germany.

The original article has been corrected.

