# Supplementary material for: Robust hand tracking for surgical telestration
Source: Int J Comput Assist Radiol Surg. 2022 May 27;17(8):1477–86. doi: 10.1007/s11548-022-02637-9 (PMC9307534; doi:10.1007/s11548-022-02637-9)
Supplement: Supplementary file 3 — (pdf 1842 KB) [file 11548_2022_2637_MOESM3_ESM.pdf]

Appendix A Supplementary Material

A.1 Model selection study

The study was performed on the validation data set described in sec. 2.1. For coarse hand localization (i.e. bounding box detection) we found that recent models like YOLOv5s, YOLOv5m [11] and Faster R-CNN [27] performed similarly. Since our downstream tasks benefit from a fast bounding box prediction, we chose YOLOv5s based on the inference time (YOLOv5s 16 ms, YOLOv5m 70 ms, Faster R-CNN 88 ms). For the skeleton tracking task, EfficientNet B2/3 [13, 14] and ResNet 34/50 [14, 28] models have been selected for this study due to their high performance in the ImageNet classification benchmark and their short inference time. Other performant models, such as vision transformers were disregarded because of their longer inference time. For the hand segmentation task we validated several models with/without auxiliary keypoints. We selected two architectures common in semantic segmentation, namely Feature-Pyramid-Network [18, 29] and U-Net [29, 30]. As backbones we used EfficientNet models B1 and B3 [13] with Noisy Student[15] pretrained weights.

	successf. loc.		regr. dist. [px]		t [ms]
	mean	iqr	mean	iqr	
Architecture					
EfficientNet B2	0.98	0.03	9.6	2.6	12
EfficientNet B3	0.98	0.03	9.3	2.1	12
ResNet 34	0.98	0.03	11.3	2.8	4
ResNet 50	0.98	0.03	10.1	2.7	4

Table A1 Skeleton tracking performance for different pipeline components We compared the popular backbone EfficientNet (B2 and B3) as well as the ResNet both with Noisy Student pretrained weights.

	backbone	aux. kp	DSC		t [ms]
			mean	iqr	
Architecture					
FPN	EfficientNet B3	False	0.95	0.02	11
FPN	EfficientNet B3	True	0.95	0.01	65
UNet	EfficientNet B1	True	0.94	0.01	55
UNet	EfficientNet B1	False	0.95	0.01	10

Table A2 Segmentation performance for different pipeline components We used a Feature-Pyramid-Network (FPN) or U-Net architecture with backbones EfficientNet B3 and B1. For both variants auxiliary keypoints (aux. kp) where used or disabled.

Springer Nature 2021 LaTeX template

A.2 Supporting figures

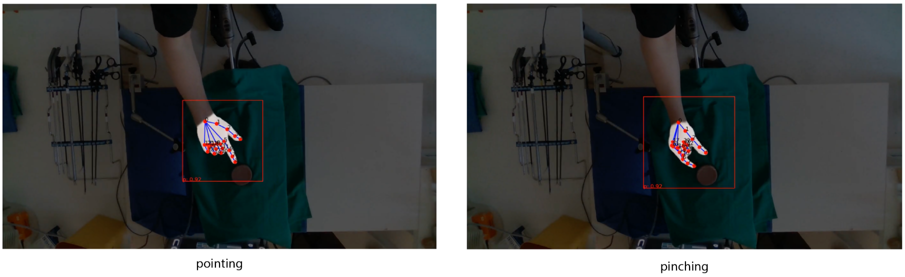

Fig. A1 Sample frames for surgical gestures. The depicted frames represent the gestures pointing and pinching. The prediction of the segmentation and skeleton tracking model is shown.

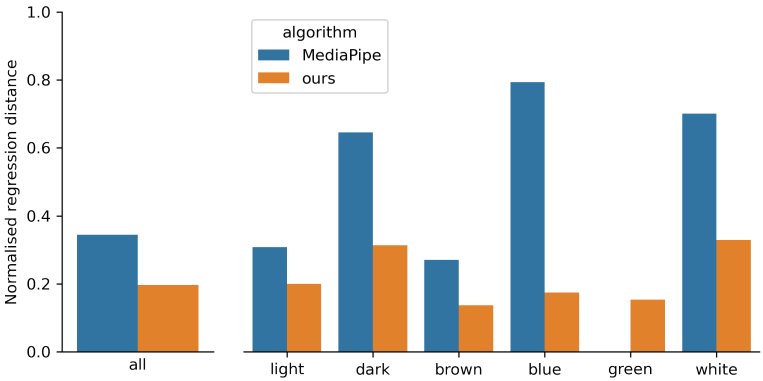

Fig. A2 Normalised mean regression distance for successful localizations. Distances were normalised by the palm breadth of the annotation (i.e. the distance between keypoint 5 and 17).

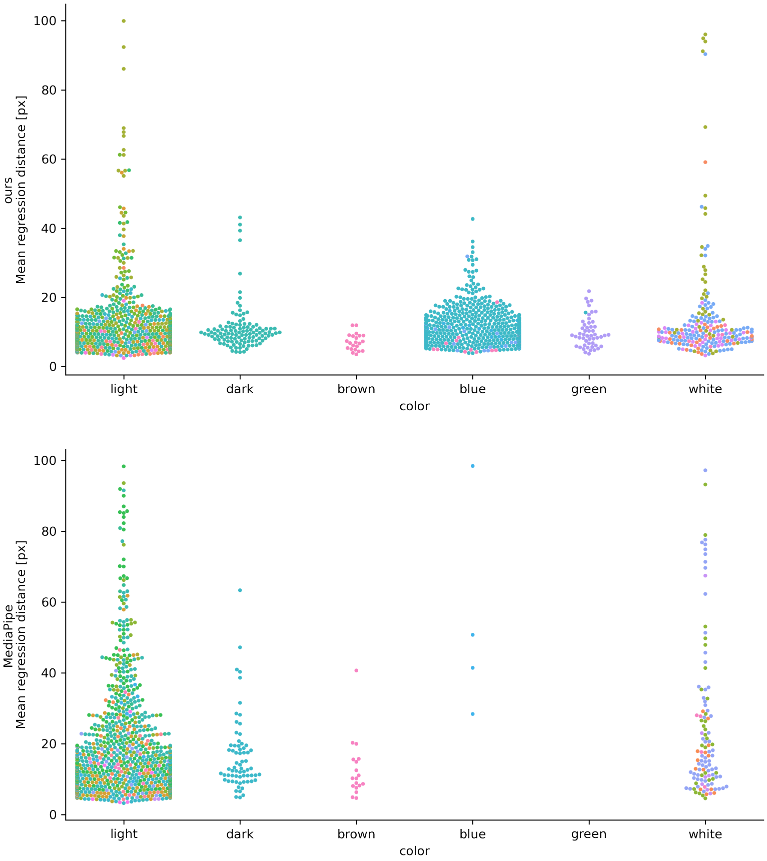

Fig. A3 Skeleton tracking performance for successful localizations as a function of hand/glove color. Each dot represents the mean regression performance on one frame of the validation data set presesented in sec 2.1. Dots with the same color correspond to the same recording day. Due to the high number of samples there is an overlap for small regression distances.
